# Relationship between Serum Soluble Interleukin-2 Receptor and Renal Allograft Rejection: A Hospital-Based Study in KashmirValley

**Published:** 2015-02-01

**Authors:** R. Rasool, Q. Yousuf, K. Z. Masoodi, I. A. Bhat, Z. A Shah, I. A. Wani, M. S. Wani

**Affiliations:** 1 Department of Immunology and Molecular Medicine,; 2 Department of Nephrology, and; 3 Department of Urology, Sher-I-Kashmir Institute of Medical Sciences

**Keywords:** Creatinine, Receptors, interleukin-2, Allografts, Graft rejection, Kidney transplantation, Immunosuppression

## Abstract

Background: Even after adequate immunosuppression therapy, acute rejection continues to be the single most important cause of graft dysfunction after renal transplantation. Renal allograft biopsy continues to be the reference standard, though certain clinical and biochemical parameters are helpful in assessment of these patients. Renal allograft rejection is mediated by T lymphocytes, expressing cell surface interleukin-2 receptors (IL-2R) which has been suggested as a marker of acute rejection episodes after organ transplantation.

Objective: To determine the pre- and post-transplantation serum soluble IL-2R levels in live related kidney transplant patients to predict acute rejection episodes.

Methods: Serial serum samples from 75 recipients and 41 healthy controls were assessed for soluble IL-2R levels by ELISA. The outcome of the graft was also determined for each recipient.

Results: The mean±SD serum soluble IL-2R levels in renal allograft recipients with rejection were significantly (p<0.001) higher than those without rejection (329.85±59.22 *vs *18.12±11.22 pg/mL). The elevation of serum soluble IL-2R was evident in acute rejection episodes and found before elevation of serum creatinine. The higher values of serum soluble IL-2R in the rejection group were significantly reduced after recovery of allograft function by adequate anti-rejection therapy. 36.4% of patients in the rejection group had proven positive biopsies for the rejection and higher creatinine values, which was found to be statistically significant (p<0.001). A cohort of 41 healthy controls showed significantly (p<0.05) lower serum soluble IL-2R concentrations (15.27±7.79 pg/mL) when compared with the rejection group.

Conclusion: Serum soluble IL-2R concentrations showed significant correlation with the acute rejection episodes in the renal allograft recipients. Prediction of soluble IL-2R levels might help the early detection of rejection episodes, which may pave way for the management of immunosuppression regimes and better graft functioning.

## INTRODUCTION

The molecular and biochemical characterization of the important lymphokine, interleukin-2 (IL-2), has been shown to occupy a pivotal role in the generation of the immune response [1,2]. IL-2 is produced by activated T cells and plays a pivotal role in the proliferation of T lymphocytes after antigenic stimulation [3]. Upon activation, the T cell expresses high-affinity receptors for IL-2 (IL-2R), and subsequently, a soluble form of the IL-2R protein (sIL-2R, 45 kDa) is released [3]. Concentrations of sIL-2R have been suggested as a marker of rejection episodes after organ transplantation [4,5]. Renal transplantation offers a definitive therapeutic modality for patients with end-stage renal disease; however, 50% to 70% of these patients suffer graft dysfunction after transplantation [6]. Despite potent immunosuppression, acute rejection continues to be the single most important cause of graft dysfunction in majority of patients. Cyclosporine (CsA) toxicity, acute tubular necrosis (ATN) and infections may also contribute to the causation of graft dysfunction in some of these patients [6]. Acute rejection of the kidney allograft is clinically defined as an elevation in the level of serum creatinine by more than 0.3 mg/dL and is diagnosed by kidney biopsy [7]. Acute renal allograft rejection is mediated by T lymphocytes; T cells expressing cell surface IL-2Rs were found in the kidneys of renal allograft recipients [8]. sIL-2R assay may be useful clinically in the differential diagnosis of renal allograft rejection, especially in distinguishing CsA nephrotoxicity [9]. In kidney-transplant recipients, sIL-2R is greatly increased during rejection or viral infections, whereas patients with CsA nephrotoxicity had sIL-2R concentrations significantly below those of patients with rejection [9]. Although elevated sIL-2R levels are encountered in patients with renal allograft rejection, a considerable variation in these levels is encountered. Due to this variation, the significance of elevated sIL-2R levels is incompletely understood [5]. To evaluate the analytical performance of a “sandwich-type” enzyme immunoassay method for sIL-2R and to verify whether increased concentrations of sIL-2R might be a useful marker of allograft rejection, we quantified sIL-2R in serum samples from kidney-transplant patients and healthy controls. 

## PATIENTS AND METHODS

We studied a cohort of 75 live related renal transplant patients who underwent transplantation at the Sher-i-Kashmir Institute of Medical Sciences (SKIMS) and other hospitals in northern India, and 41 healthy controls. The blood samples of all participants were collected from the Department of Nephrology after the approval from the SKIMS Ethical Clearance Committee and taking a written informed consent from the patients. Serum was isolated by standard centrifugation. Blood samples were taken at various times—within five days of transplantation, at one month, and three months. Samples were stored at –70 °C for further use. The graft rejection was clinically diagnosed by rise in serum creatinine levels, graft tenderness, increased body weight, decreased urine output, and diethylene triamine penta-acetic acid (DTPA) scan. However, the renal allograft biopsy continues to be the reference standard. Serum sIL-2R values were correlated with the occurrence of graft rejection on histology.

Serum sIL-2R levels were dynamically measured with enzyme linked immunosorbent assay (ELISA) in patients with renal allografts and in healthy controls using a commercially available Human IL-2 ELISA kit (Gen-Probe, Diaclone, France) as per the manufacturer’s instructions. The concentration reference range of sIL-2R ranges from 1–1000 (pg/mL). Serum quantification of sIL-2R was also determined in 41 healthy volunteers without any acute or chronic medical illness. The serum creatinine level was used as a parameter of post-transplant graft function.

## RESULTS

**Figure 1:**
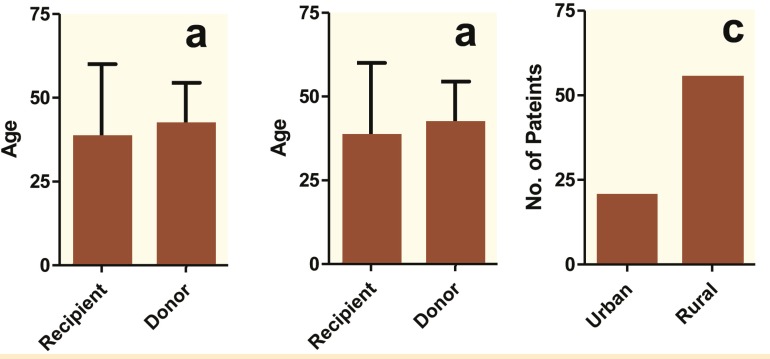
Comparative case-control analysis for age, gender and dwelling of kidney transplant patients. a) Age of donor and recipients for the kidney transplant. b) Patients categorized according to their gender. c) Distribution of patients according to their residence—rural or urban areas.

The clinical information including gender, age, disease history, dwelling, biochemical investigations and treatment charts were obtained from the review of patients/medical records (Fig 1, Table 1). There were 67 males and 8 females with an age at the transplant time ranging from 16 to 65 (mean 38) years. The cohort of patients was predominantly from rural areas (n=55) in comparison to the urban area (n=20) (Fig 1). 

**Table 1:** Baseline clinical characteristics of studied kidney transplant recipients (n=75).

Parameter	Mean±SD or n (%)
Recipient age (yrs)	38.3±21.8
Donor age (yrs)	42.31±12.2
Recipient Gender	
Male Female	67 (89%) 8 (11%)
Creatinine (mg/dL)	3.64±1.96
Blood Urea Nitrogen (mg/dL)	48.33±35.68
Recipient dwelling	
Rural Urban	55 (73%) 20 (27%)
Hemoglobin (g/dL)	12.2±2.6

**Figure 2:**
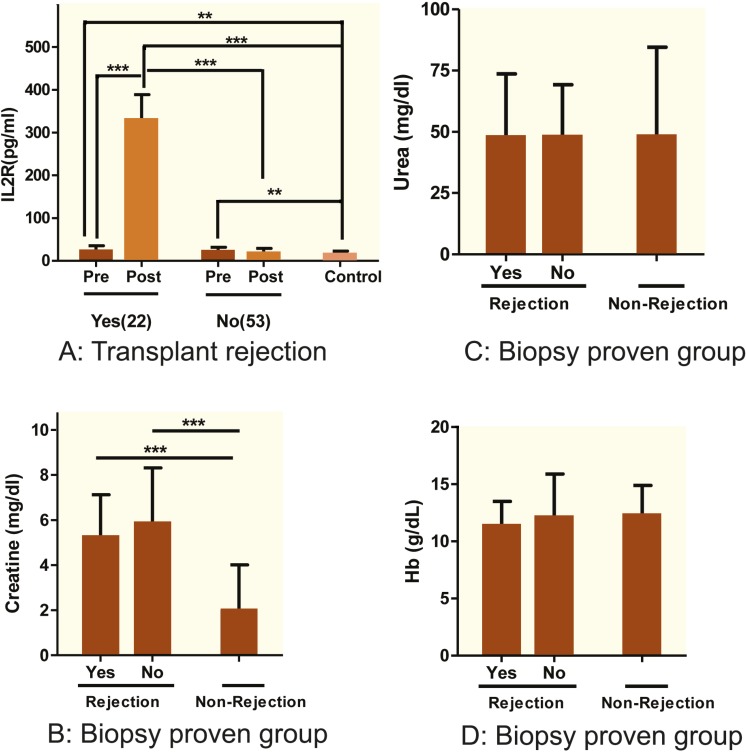
Temporal courses of cytokine levels and clinical parameters in kidney transplant patients. A) IL-2R levels (pg/mL) in pre- and post-kidney transplant patients with (n=22) and without (n=53) graft rejection. Data represents 75 kidney transplant patients and 41 healthy controls. B) Comparative analysis of creatinine levels in male and female kidney transplant recipients. The rejection group comprised of both patients who did have and not have proven kidney biopsy. C) Blood urea nitrogen (BUN, mg/dL) was measured in patients with (n=22) and without (n=53) graft rejection. D) Comparative hemoglobin levels (g/dL) in kidney transplant recipients; 75 cases and 41 controls were used in the evaluation.

All the renal allograft recipients were divided into two groups: those with rejection and those without, depending on the levels of the sIL-2R in the sera of these patients (Table 2). The rejection group included 22 (29.3%) patients (8 biopsy-proven), while the non-rejection group included 53 (70.7%) patients (Fig 2A). During the post-transplantation period, rejection group had a significantly (p<0.001) higher mean±SD sIL-2R level (329.85±59.22 pg/mL) compared to the non-rejection group (18.12±11.22 pg/mL). There was no rise in sIL-2R levels in 14 (64%) patients who were clinically showed signs of rejection episodes (evident by their higher creatinine levels) that not confirmed by renal biopsies. The recipients with graft rejection showed increased sIL-2R levels (mean**±**SD of 329.85±59.22 pg/mL) when compared with the pre-transplant patients (23.56±2.52 pg/mL) (p<0.001). sIL-2R levels in pre-transplant group (mean**±**SD of 23.56±2.52 pg/mL) increased significantly (p=0.004) in comparison to the healthy controls (15.27±7.79 pg/mL). Post-transplant graft rejection group also showed an increased serum sIL-2R levels compared to the healthy controls (p<0.001). Serum creatinine levels was significantly (p<0.05) higher in patients who had acute graft rejection (mean±SD of 5.27±1.86 mg/dL) compared to those with no history of rejection (2.02±1.99 mg/dL) (Fig 2B). The mean±SD blood urea nitrogen levels in those with acute rejection (48±25.63 mg/dL) was not significantly different from that in those without rejection (48.35±36.18 mg/dL) (Fig 2C). There was also no significant difference in hemoglobin levels between the rejection and the non-rejection groups.

**Table 2:** Serum sIL-2R levels in renal allograft recipients with rejection episodes compared with the patients having no episodes of rejection.

Variable	Rejection group (n=22)		Non-Rejection group (n=53)	p Value
	Biopsy-proven (n=8)	Not proven by biopsy (n=14)		
Control (pg/mL)	NA*	NA	15.27±7.79	—
Pre-transplant (pg/mL)	23.56±11.83	24.12±7.65	21.16±10.70	0.31
Post-transplant (within 1-3 month) (pg/mL)	329.85±59.22	28.37±10.31	18.12±11.22	0.0001
BUN (mg/dL)	48±25.63	48.22±21	48.35±36.18	0.97
Creatinine (mg/dL)	5.27±1.86	5.89±2.43	2.02±1.99	0.0001
Hemoglobin (g/dL)	11.36±2.13	12.13±3.77	12.28±2.61	0.0001
Dwelling				
Rural	3	11	37	
Urban	5	7	12	0.078
Mean±SD recipient age (yrs)	38.7±3.1	38.3±7.9	38.3±10.1	0.9473
Gender				
Male	8	12	47	0.719
Female	0	2	6	

* NA: Not applicable

Significance level (*Student’s t* test): *p<0.05; **p<0.01; ***p<0.001.

Biopsy of the allograft kidney in those who were declared to have rejection showed significant interstitial infiltration, foci of moderate tubulitis (5–10 mononuclear cells per tubular cross section), and features of segmental sclerosis in the glomerulus. The glomeruli revealed presence of few intra-capillary mononuclear and polymorphonuclear cells suggesting T-cell-mediated rejection. In addition, histochemical staining for C4d showed diffuse positivity (n=5) along the peritubular capillaries suggesting antibody-mediated rejection. Evidence of diffuse severe acute tubular injury with luminal sloughed epithelial cells and few hyaline casts in tubular lumina were noted in some patients. These patients regained normal graft function after the anti-rejection therapy was given. However, one of the patients with acute rejection showed rising in serum creatinine level and did not respond to the anti-rejection therapy and died.

## DISCUSSION

Activation of T-lymphocytes in response to alloantigen is a central component of the rejection process after organ transplantation. Therefore, in the absence of infection, one could assume that increased sIL-2R levels might be a tool to evaluate the presence of rejection activity [10]. Indeed, regarding sIL-2R production in kidney recipients, it has been demonstrated that serum levels of sIL-2R are significantly higher in patients suffering renal allograft rejection compared to patients with stable graft function and that the serial evaluation of serum sIL-2R increases the sensitivity and specificity of the test [10]. The increase of serum sIL-2R has also been shown to be comparable to the rise in serum creatinine values observed in rejection episodes with the predictive value of the combined tests being superior to either alone [11]. 

In our study, we found that patients experiencing renal allograft rejection had a significantly higher concentration of serum sIL-2R after transplantation compared to those without rejection. The higher levels of sIL-2R found in patients experiencing rejection may reflect T cell activation by the allografts. Such high levels of sIL-2R have also been reported before that supports the notion that IL-2R can be used as a diagnostic marker for graft rejection in kidney transplant patients [12]. In the present study, none of the patients in the rejection group had any other causes of graft dysfunction such as infection, which was ruled out by blood and urine culture with radiology, or CsA toxicity ruled out by measuring the drug levels. The higher values of sIL-2R in the rejection group were significantly reduced after recovery of the allograft function by adequate anti-rejection therapy including pulses of steroid therapy and other immunosuppressive drugs. The healthy control group showed lower sIL-2R concentrations. The elevation of serum sIL-2R was evident in acute rejection episodes and was found before the elevation of serum creatinine; 36.4% of patients with acute rejection experienced renal allograft rejection with proven biopsies and higher creatinine values. Therefore, our study demonstrated that serum sIL-2R levels in renal allograft recipients might pave the way for the early detection of the rejection episodes, which can be treated by adequate drug regimen modifications or replacement therapy. 

In conclusion, sequential monitoring of sIL-2R in association with other clinical markers may predict acute rejection of renal allograft recipients without invasion. Early detection of rejection may help clinicians in applying appropriate management that would result in better graft functioning.
